# Cinnamaldehyde/cinnamon essential oil loaded poly-L-lactic acid/ hydroxyapatite fibrous scaffolds as osteogenic differentiation enhancing system for bone tissue engineering applications

**DOI:** 10.1186/s13036-025-00547-3

**Published:** 2025-09-30

**Authors:** Fateme Darchin Tabrizi, Mehdi Doosti-Telgerd, Narges Forouzideh, Fatemeh Naghshnejad, Ehsan Seyedjafari, Hamid Akbari Javar, Fatemeh Saadat Mahdavi, Mina Habibizadeh, Mahdi Tavakolizadeh

**Affiliations:** 1 Department of Nanotechnology or Pharmacognosy, Faculty of Pharmacy, Zanjan University of Medical Sciences, Zanjan, Iran; 2 Department of Pharmaceutics, School of Pharmacy, Medicinal Plants and Natural Product Research Center, Hamadan University of Medical Sciences, Hamadan, Iran; 3 Departments of Pharmaceutical Nanotechnology, School of Pharmacy, Zanjan University of Medical Sciences, Zanjan, Iran; 4 Department of Cell and Developmental Biology, School of Biology, College of Science, University of Tehran, Tehran, Iran; 5 Departments of Biotechnology, College of Science, University of Tehran, Tehran, Iran; 6 Departments of Pharmaceutics, Faculty of Pharmacy, Tehran University of Medical Sciences, Tehran, Iran; 7 Regenerative Medicine Research Center, Kermanshah University of Medical Sciences, Kermanshah, Iran; 8 Departments of Pharmacognosy, School of Pharmacy, Zanjan University of Medical Sciences, Zanjan, 45139-56184 Iran; 9 Evidence-based Phytotherapy and Complementary Medicine Research Center, Alborz University of Medical Sciences, Karaj, Iran

**Keywords:** Poly-L-Lactic acid, Hydroxyapatite, Fiber, Osteogenesis, Cinnamaldehyde, Cinnamon essential oil, Bone tissue engineering

## Abstract

In this study, we developed electrospun poly-L-lactic acid (PLLA)/ hydroxyapatite (HA) fibers loaded with Cinnamaldehyde (Cin) or Cinnamon essential oil (E.O) as a dual-function system to modulate bone remodeling. Cin or E.O as a natural component inhibited the bone resorption same as the bone formation process. The electrospun fibers were characterized by SEM, FTIR, contact angle, and tensile test. The biocompatibility of scaffolds was evaluated by an MTT assessment. Additionally, the osteogenic potency of fibers was investigated by alkaline phosphatase (ALP) activity assay, Alizarin Red staining, calcium content assay, and the RT-PCR analysis. The cinnamon compounds release profile was studied over 12 days. The results demonstrated that PLLA/HA/Cin and PLLA/HA/E.O. fibers had bead-free, randomly oriented morphologies and suitable physicochemical properties for cell seeding and survival. The calcium level deposition and ALP activity of PLLA/HA/Cin and PLLA/HA/E.O significantly increased compared to the control group. Osteogenic-related gene expression including ALP, Runx2, osteocalcin, and osteonectin significantly upregulated in PLLA/HA/Cin fiber group. The results revealed that the PLLA/HA/Cin fiber with desirable osteogenic differentiation potency can be an appropriate candidate for bone tissue engineering applications.

**Graphical abstract:**

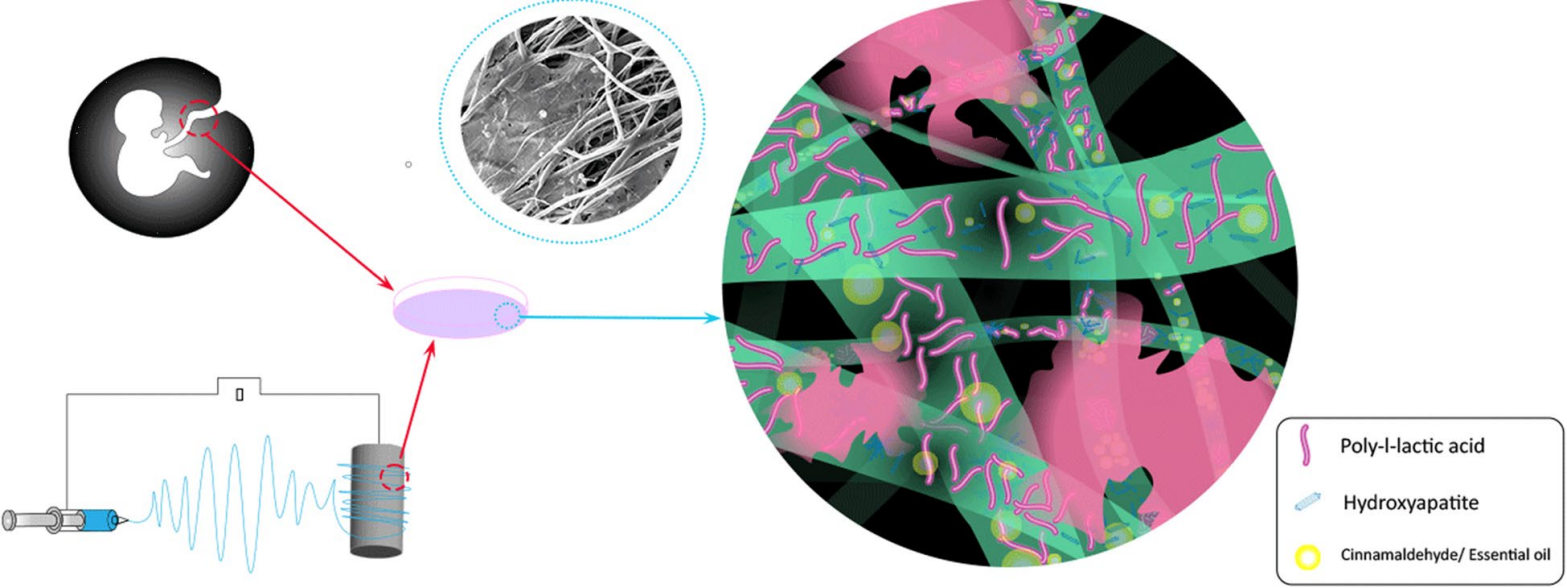

## Introduction

Bone remodeling is a long-life process, which involves bone resorption by osteoclasts followed by bone formation, where different cytokines regulate this two-way process. Therefore, treatment of bone injuries usually faces clinical complications [1]. Bone tissue engineering as a multidisciplinary science has used the principles of engineering and biological sciences to improve, maintain, or repair damaged bone tissue by fabricating biodegradable scaffolds, using biomimetic approaches, and distributing cells on their surfaces [2, 3]. Construction of an appropriate scaffold that can have proper biological and mechanical properties is one of the concerns of tissue engineering (TE) that is important for stimulating the damaged tissue, growth, and differentiation of cells, and finally regeneration of damaged tissue [4]. To the best of our knowledge, some researchers designed and developed multifunctional scaffolds that have a synergistic impact due to the employment of two biomolecules that modulate the balance of bone formation and bone resorption [5]. Designing a multifunctional fibrous scaffold by employing one natural biomolecule which in itself has a two-way effect of bone remodeling as a differentiation enhancing system is more convenient and economical to repair bone defects as rapidly and effectively as possible. According to the findings of other researchers, electrospun poly-L-lactic acid (PLLA)/ hydroxyapatite (HA) fibers are one of the most attractive fibers for bone tissue engineering due to their ability to promote bone formation [6]. PLLA is a common synthetic biocompatible and biodegradable polymer that is approved by the FDA for various biomedical applications such as TE [7, 8]. While the addition of nanoscale mineral particles into a PLLA fibrous structure can improve the biomineralization capacity of the fibrous scaffold, which is necessary for cellular integrations, due to increasing material surface stiffness without reducing its mechanical strength [9]. HA is a hydrophilic mineral compound, employed to enhance cell adhesion by improving their biocompatibility and functionality [10]. HA nanoparticles due to their structural features, size in the nano dimension, and morphology, can increase the absorption of proteins and the adhesion of cells in the scaffold [11, 12].

Cinnamaldehyde (Cin) is a natural aldehyde compound extracted from cinnamon bark that has been proven to be useful for different functions such as anti-bacterial and anti-inflammatory effects and the inhibition of inflammatory cytokines by other researchers. It is confirmed by both in vitro and in vivo that Cin plays an important role in the treatment of osteoporosis by enhancing the promotion of osteoblasts and inhibition of osteoclasts [13–17].

Tsuji-Naito et al.. found that Cin as an aldehyde component of *Cinnamomum zeylanicum* bark extract inhibits the receptor activator of nuclear factor-ĸB ligand (RANKL) induced nuclear factor of activated T Cell 1 (NFATc1) which has a crucial rule for osteoclastogenesis [18, 19]. In addition, Zongyi wua et al. found that Cin stimulates ossification on the distal femur in ovariectomized rats. Additionally, recent studies have indicated that Cin can act as an agonist of nuclear factor erythroid 2-related factor (Nrf_2_) which can reduce hydrogen peroxide (H_2_O_2_) exposure by mitochondrial dysfunction and thus enhance the osteogenic differentiation of bone mesenchymal stem cells [20].

In previous studies, the use of cinnamon (Cin) or cinnamon essential oil (E.O.) for inhibiting osteoclastogenesis and stimulating osteogenesis has been performed without the incorporation of any scaffolding. This limitation leads to the rapid release of Cin and E.O., which is a drawback given that bone remodeling is a lifelong process. Observing differentiation without scaffolding requires daily treatment with Cin or E.O. To address this issue, employing electrospun fibers for local administration and controlled release of Cin or E.O. represents an effective strategy for sustained enhancement of mesenchymal stem cell (MSC) differentiation.

In this study, we designed and developed an electrospun PLLA/HA fibrous scaffold for the delivery of Cin or E.O. to achieve a synergistic effect, enhancing both bone resorption and bone formation to support MSC differentiation and proliferation. The osteogenic performance of Cin or E.O.-loaded PLLA/HA fibers was evaluated using Wharton’s jelly MSCs in an in vitro model, compared to control groups with PLLA/HA and PLLA scaffolds. While several reports have explored the osteogenic potential of cinnamaldehyde or cinnamon essential oil in solution, these studies typically lack a delivery platform capable of providing sustained, localized release and structural support.

Our study uniquely integrates these natural bioactives into electrospun PLLA scaffolds enhanced with HA, enabling a dual-function system that promotes osteogenic differentiation while potentially modulating bone resorption. This synergistic combination of bioactivity and biomimetic architecture represents a novel strategy for bone tissue engineering applications. These points are now explicitly stated in the revised Introduction section.

## Materials and methods

### Materials

PLLA, fetal bovine serum (FBS), dexamethasone, ascorbic acid, β-glycerophosphate, chloroform, and Cin were purchased from Sigma-Aldrich; Germany. Dimethyl sulfoxide (DMSO) was purchased from Life Biolab; England. Minimum essential medium (MEM), alpha modifications, and sodium bicarbonate were purchased from Gibco; England. HA nanoparticle was purchased from NanoSadra Co; Iran. The alkaline phosphatase (ALP) activity Assay kit and calcium content assay kit was purchased from Pars Azmun Co; Iran. E.O was purchased from Zardband Pharmaceutical Co; Iran.

### Preparation of fibers

To prepare Cin and E.O-loaded fibers, various concentrations of Cin and E.O. (1–1000 µg/mL) were first evaluated for cytotoxicity and cell differentiation using MTT and calcium content assays. Based on these results, the optimal concentrations (900 µg/mL of Cin and 600 µg/mL of E.O.) were selected for fiber preparation. The detailed methodologies are described below.

#### Preparation of PLLA and PLLA/HA fibers

PLLA fiber was prepared by electrospinning as follows: 0.6 g of PLLA (10% W/V) was dissolved in 6.0 ml of chloroform while stirring overnight, to result in a clear solution. Then, 1.0 ml DMF was added to the solution and stirred until a transparent solution without bubbles was formed. The final solution was electrospinning by these parameters; 19 kV voltages, 0.5 ml/h flow rate, 8 cm nozzle-to-collector distance, and 600 rpm collector speed.

For PLLA/HA composite fiber preparation, 0.3 g of HA nanoparticles in 25 nm size (5% W/V) was dispersed in 1.0 ml DMF with ultrasonic bath (30 min, 30 °C), then was added to 10% W/V PLLA solution and was stirred to prepare a uniform suspension and then was employed for electrospinning similar to the previous method [21].

#### Preparation of cin and E.O loaded fibers

To prepare the loaded PLLA fiber, 900 µg/ml of Cin (0.9% w/w) and 600 µg/ml of E.O (0.6% w/w) were dissolved in 1.0 ml of DMF separately. Then they were added to 10% W/V PLLA solution and were stirred to form a clear solution. Both final solutions were electrospinning by similar parameters as the mentioned method. To prepare composite-loaded fiber scaffolds, a prepared mixed solution of 10% w/v PLLA was added to 1 mL of 5% HA in DMF and stirred thoroughly to achieve a uniform suspension. The final suspension was then used for electrospinning, as described previously.

### Characterization

#### Characterization of fibers

The surface morphology of fiber scaffolds was evaluated by scanning electron microscope (SEM). Some pieces of all scaffolds were coated with a thin layer of gold and imaging was done at different magnifications with SEM (NOVANANOSEM 450; USA). The diameter of the fibers was measured from the SEM micrographs using image analysis software (Image J, National Institutes of Health; USA). The presence of components in the composited fibers was analyzed by Fourier transform infrared spectrometer (FT-IR, Perkin Elmer Frontier; USA). The FTIR spectra in the transmission mode were recorded using the FTIR spectrometer (Bruker, Tensor 27 FTIR Spectrometer; USA) in the range 4000–400 cm^− 1^ and the data was analyzed by OPUS 6.0 software. The tensile properties of fibers were evaluated by a tensile test machine (Santam tensile machine, STM 20; Iran). For this purpose, all the samples of the scaffolds were cut into 4 × 1 cm and placed in the tensile tester. The force was applied with a speed of 5 mm/min until rupture occurred at room temperature.

#### Plasma treatment and surface hydrophobicity measurement

Plasma treatment was used to improve the hydrophilicity of the surface of scaffolds. For this purpose, a low-frequency (40 kHz) generator with 30 w powers was used inside a quartz cylindrical reactor. The vacuum was applied, and then high-purity oxygen gas flowed in the chamber for 10 min until uniform its atmosphere was complete. The samples were placed in the chamber to treat the surface fibers with UV rays. Finally, samples were exposed to air by breaking the vacuum.

To evaluate the hydrophilicity of the fibers, the contact angle was measured before and after plasma treatment. To do this, a drop of water was poured on the surface of the fiber and it was photographed. The contact angle of the drop and the fiber’s surface was determined by ImageJ software. Finally, the yield data graph was plotted with GraphPad Prism and analyzed with an unpaired t-test.

#### Release profiles of cin and E.O

The dialysis method was used for the release study. In brief, each sample of fibers was placed in a dialysis bag and closed with a strong thread after adding PBS solution. Dialysis bags were placed in falcons with 20 ml of PBS containing 20% (v/v) ethanol with pH 7.4. All samples were placed in a shaking incubator at 37 °C temperature on predetermined time points (15 min, 40 min, 1 h, 2 h, 4 h, 8 h, 12 h, 24 h, 48 h, 96 h, 144 h, and 288 h), 1 ml of the release media were collected and replaced by a refresh medium buffer. The concentration of the release samples in the medium was obtained by UV-VIS spectrophotometer at 283 nm. All the release studies were studied in triplicate. Finally, the cumulative drug release percentage was calculated and the release profile was drawn with GraphPad Prism 8.

### Cell experiments preparations

#### Cell culture and material sterilization

##### Cell proliferation and adhesion

To evaluate the morphology and adhesion of cells onto the surface of PLLA, PLLA/HA, PLLA/Cin, and PLLA/HA/E.O fibrous scaffolds, the samples were sterilized using ethanol 70% and UV, followed by washing with PBS on 24-well plates. Wharton’s jelly cell suspension at a density of 5 × 10^3^ cell density/cm^2^ in MEM alpha medium supplemented with 10% v/v FBS as the basal medium was seeded on fibers. The tissue culture plates were then incubated. Seven days after seeding, the cells were fixed on the surface of the fibers. Cell morphology and attachment were subsequently examined by SEM.

### Evaluation of cell viability by MTT assay

#### Cell viability and IC_50_ evaluation of cin and E.O

In vitro, cytotoxicity evaluation of Cin and E.O was performed by MTT assay. For this purpose, Wharton’s jelly cells (passage 4, 5 × 10^3^ cell density/cm^2^) in MEM alpha medium supplemented with 10% v/v FBS were seeded in 96-well plates and incubated at standard conditions including 5% CO_2_ and 37 °C temperature. Treatment groups included various concentrations of Cin and E.O (1–1000 µg/ml) and a control group, with all treatments prepared in the supplemented medium. After one day the culture medium was replaced with a treatment medium. Following 24 h of incubation, the cells were treated with MTT reagent (150 µL, 5 mg/mL) and incubated for 4 h at 37 °C and 5% CO_2_. Subsequently, DMSO was replaced for dissolving formazan crystals, and the absorbance of the resulting solution was measured using a microplate reader at 570 nm. Cell viability was normalized to the control group, and the IC_50_ values for both Cin and E.O. were determined.

#### Biocompatibility assessment of fibrous samples

To evaluate the cytotoxicity of fibers, the samples were sterilized using ethanol 70% and UV, followed by washing with PBS in 48well plates. Wharton’s jelly cells (passage 4, 1.5 × 10^4^ cell density/cm^2^, purchased from Royan stem cell technology Co.) were seeded onto the fibers in MEM alpha medium supplemented with 10% FBS as the basal medium. The plates were then incubated at 37 °C with 5% CO_2_. MTT assays were performed at specified time points (1, 3, and 7 days after cell seeding) as previously described.

### Differentiation impact of cin and E.O

To evaluate the effect of Cin and E.O. on the differentiation of MSCs, a calcium content assay was performed. For this purpose, cell suspension containing 3 × 10^4^ cell density/cm^2^ of Wharton’s jelly cells in passage 4 with MEM alpha medium was seeded on 48 well plates and incubated at standard conditions including 5% CO_2_ and 37 °C temperature. The treatment groups included various concentrations of Cin and E.O (1–1000 µg/ml), prepared in MEM alpha supplemented with 10% FBS, 1% β-glycerophosphate, 1% ascorbic acid, and 0.1% dexamethasone. The control group contained only the supplemented medium.

The culture medium was replaced with the treatment medium one day after seeding, and the fresh supplemented medium was added every three days. A calcium content assay was performed on days 7 and 14 after treatment. For the assay, each well was washed with PBS, followed by the addition of 0.6 N HCl. The resulting samples were processed using a calcium assay kit, and the absorbance was measured at 570 nm using a microplate reader.

### Differentiation effect of fibers

To evaluate the osteogenic differentiation potential of fibers, a cell suspension containing 1.5 × 10^4^ cell density was seeded onto the sterilized fibers. A control group without any fibers was considered for comparison. MEM alpha was used as a basal medium supplemented with 10% FBS, 1% β-glycerophosphate, 1% ascorbic acid, and 0.1% dexamethasone. Then well plates were incubated to perform differentiation tests.

#### Alizarin red staining study

The Alizarin Red staining method was used to evaluate the osteogenic differentiation of cells on fibrous samples at days 7, 14, and 21. For this purpose, the cells were fixed on the samples with glutaraldehyde 2.5% V/V after washing with PBS. Dehydration was performed sequentially using 50%, 70%, and 100% ethanol. Then, Alizarin Red solution was added to fibers, and the well plates were incubated for 3 min. Finally, the samples were washed with PBS, and differentiated cells were imaged by a stereo microscope.

#### Calcium content study

The calcium content of cells was determined by a calcium assay kit to evaluate the osteogenic differentiation of Wharton’s jelly cells on fiber surfaces at days 7, 14, and 21 post-seeding. For this purpose, the fibers were homogenized in HCl 0.6 N and centrifuged for 5 min. Finally, the calcium content was determined by a calcium assay kit which is based on the interaction of cresolphthalein complex one interaction by serial dilution of standard solution in the kit. Complex absorbance was measured via a microplate reader at 570 nm.

#### Alkaline phosphatase study

ALP activity calorimetric assay kit was applied to evaluate the differentiation of Wharton’s jelly cells at osteogenic days 7, 14, and 21 after seeding on surface fibers. For this purpose, cells were lysed with radioimmunoprecipitation assay (RIPA) buffer and fibers were microfuge for 5 min. Then 40 µl of supernatants was added to kits and they were incubated for two hours. Finally, the activity of the ALP enzyme (IU/L) was normalized against the total protein (mg/dl) which was measured via a microplate reader at 405 nm.

#### Real-time PCR for gene expression

Total RNA from differentiated stem cells was extracted on days 7 and 14 after cell seeding on non-loaded fibers, Cin-loaded fibers, and a control group. Total RNA was converted to cDNA. Real-Time PCR was performed by 1X SYBR Premix Ex Taq II commercial kit, and then a thermal cycler was employed for detection. Table 1 shows the listed primer sequences. To quantify the final data, they were normalized by a β-acting housekeeping gene; standard curve method was performed by REST-RG software. Table 2 shows β-acting housekeeping gene Ct values and variability.

**Table 1:** The sequence of the primers, employed in this study

NO.	Name	Seq.(5 − 3)
**1**	H-beta Actin-F	CTT CCT TCC TGG GCA T
	H-beta Actin-R	GTC TTT GCG GAT GTC CA
**2**	osteocalcin (OSC)	GCAAAGGTGCAGCCTTTGTG
	osteocalcin (OSC)	GGCTCCCAGCCATTGATACAG
**3**	ALP-F	GCA CCT GCC TTA CTA ACT
	ALP–R	AGA CAC CCA TCC CAT CT
**4**	osteonectin (OSN) -F	AGGTATCTGTGGGAGCTAATC
	osteonectin (OSN) R	ATTGCTGCACACCTTCTC
**5**	RUNX2–F	CGGAATGCCTCTGCTGTTATG
	RUNX2–R	CTTCTGTCTGTGCCTTCTGG

**Table 2:** Average of Ct value and variation of β-acting housekeeping gene

**Day 7**	**Gene**	**Runx2**	**Osteocalcin**	**ALP**	**Osteonectin**
	TCP	17.93 ± 0.35	17.89 ± 0.17	17.98 ± 0.19	18.06 ± 0.46
	PLLA	17.51 ± 0.41	17.52 ± 0.50	17.68 ± 0.48	17.51 ± 0.41
	PLLA/Cin	18.01 ± 0.56	18.14 ± 0.61	18.70 ± 0.12	18.15 ± 0.76
	PLLA/HA	19.70 ± 1.11	20.23 ± 0.82	19.95 ± 1.35	20.03 ± 1.02
	PLLA/HA/Cin	19.01 ± 0.08	19.35 ± 0.52	19.33 ± 0.30	19.46 ± 0.41
**Day 14**	**Gene**	**Runx2**	**Osteocalcin**	**ALP**	**Osteonectin**
	TCP	17.80 ± 0.25	17.72 ± 0.33	17.63 ± 0.30	17.64 ± 0.39
	PLLA	17.64 ± 0.39	17.11 ± 0.32	17.95 ± 0.43	18.00 ± 0.44
	PLLA/Cin	20.05 ± 0.74	19.83 ± 0.20	18.77 ± 0.25	19.29 ± 0.38
	PLLA/HA	19.55 ± 0.08	19.34 ± 0.26	19.51 ± 0.70	19.14 ± 0.26
	PLLA/HA/Cin	19.65 ± 0.08	19.65 ± 0.32	20.03 ± 0.10	19.58 ± 0.37

### Statistical analysis

Statistical significance analysis was determined by GraphPad Prism 8.0 and the *p*-value ˂ 0.05 were considered statistically different. The Rest software from the rotor-gene Q, based on the Pfaffl mathematical method, was used for the statistical analysis of the real-time data and the relative gene expression. All results are reported for at least three experiments as the mean ± SD. (**P < 0.01, ***P < 0.001, ****P < 0.0001)

## Results and discussion

### Characterization of fibers

**Fig. 1:**
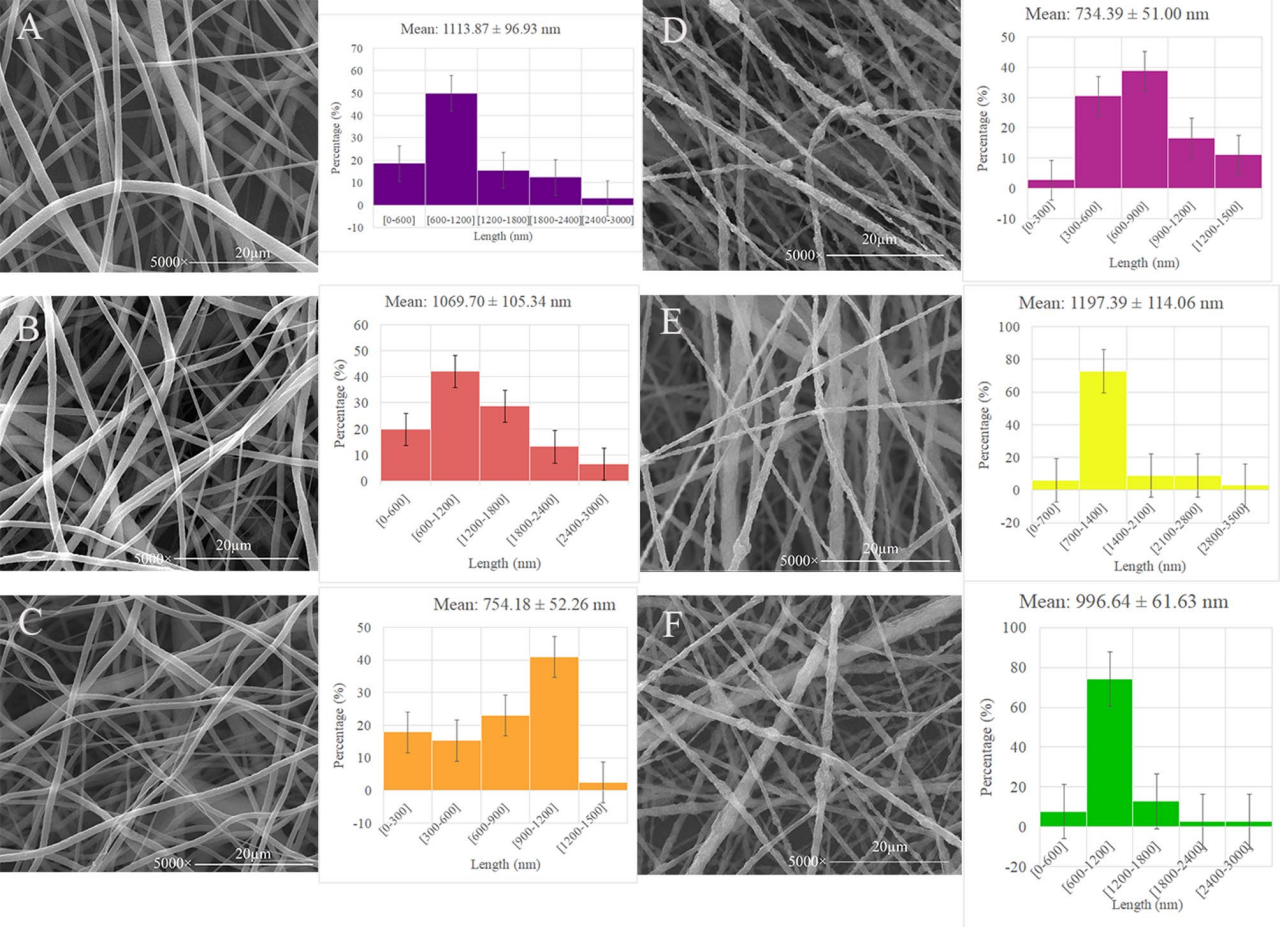
Representative SEM image and fiber diameter distribution diagrams of electrospun fibrous scaffolds included (**a**) PLLA, (**b**) PLLA/E.O, (**c**) PLLA/Cin, (**d**) PLLA/HA, (**e**) PLLA/HA/E.O, and (**f**) PLLA/HA/Cin with average diameter size of 1113.87 ± 96.93 nm, 1069.70 ± 105.34 nm, 754.18 ± 52.26 nm, 734.39 ± 51.00 nm, 1197.39 ± 114.06 nm and 996.64 ± 61.63 nm, respectively. (Mean ± SD, *n* > 3)

The morphology of PLLA (Fig. 1A), PLLA/E.O (Fig. 1B), PLLA/Cin (Fig. 1C), PLLA/HA (Fig. 1D), PLLA/HA/E.O (Fig. 1E), and PLLA/HA/Cin (Fig. 1F) fibers was analyzed using SEM. The size distribution of the average fiber diameters is shown in Fig. 1. The SEM images revealed that the fibers exhibited an appropriate morphology with uniformly and completely interconnected porous structures. Fibers had smooth surfaced without any beads or granules. The morphological analysis of fibers showed that the incorporation of HA nanoparticles into PLLA fibers reduced the average fiber diameter (PLLA and PLLA/HA fibers with average diameter sizes of 1113.87 ± 96.93 nm and 734.39 ± 51.00 nm, respectively). This reduction is likely due to changes in the viscoelastic properties of the polymer solution upon the addition of HA particles [22]. Previous studies have highlighted that fiber within the nanoscale range, along with high porosity, and uniformity of pores. Furthermore, the addition of minerals like HA to polymeric fibers has been shown to enhance their mineralization capacity [12, 23]. However, the incorporation of HA nanoparticles did not remarkably change the porosity and pore size of the fiber. On the other hand, the surface of HA-incorporated fibers became rougher compared to PLLA fibers, which may enhance the mineralization effect of the fibers [24, 25]. Also, surface of PLLA fibers was smooth that is clearly evident by SEM imaging (Fig. 1A, 1 and 1). However, HA-incorporated fibers were rough in SEM images, which are because HA nanoparticles were loaded in the surface of the fibers, not inside the fibers, so SEM imaging shows the roughness of HA nanoparticles, not beads fibers (Fig. 1.D, 1 and 1) [26–28].

In addition, the average fiber diameter of PLLA/E.O and PLLA/Cin fibers (1069.70 ± 105.34 nm and 754.18 ± 52.26 nm, respectively) reduced in comparison with PLLA fibers, maybe due to a gradual decrement in polymer viscosity by loading E.O or Cin to the polymer matrix [29]. However, the results showed that the fiber diameter increased when HA and E.O or Cin were added to the PLLA matrix simultaneously. This can be seen in PLLA/HA/E.O and PLLA/HA/Cin fibers with average diameter sizes of 1197.39 ± 114.06 nm and 996.64 ± 61.63 nm. Possibly this was due to a change in viscoelastic property or a reduction in electrical conductivity [30, 31].

**Fig. 2:**
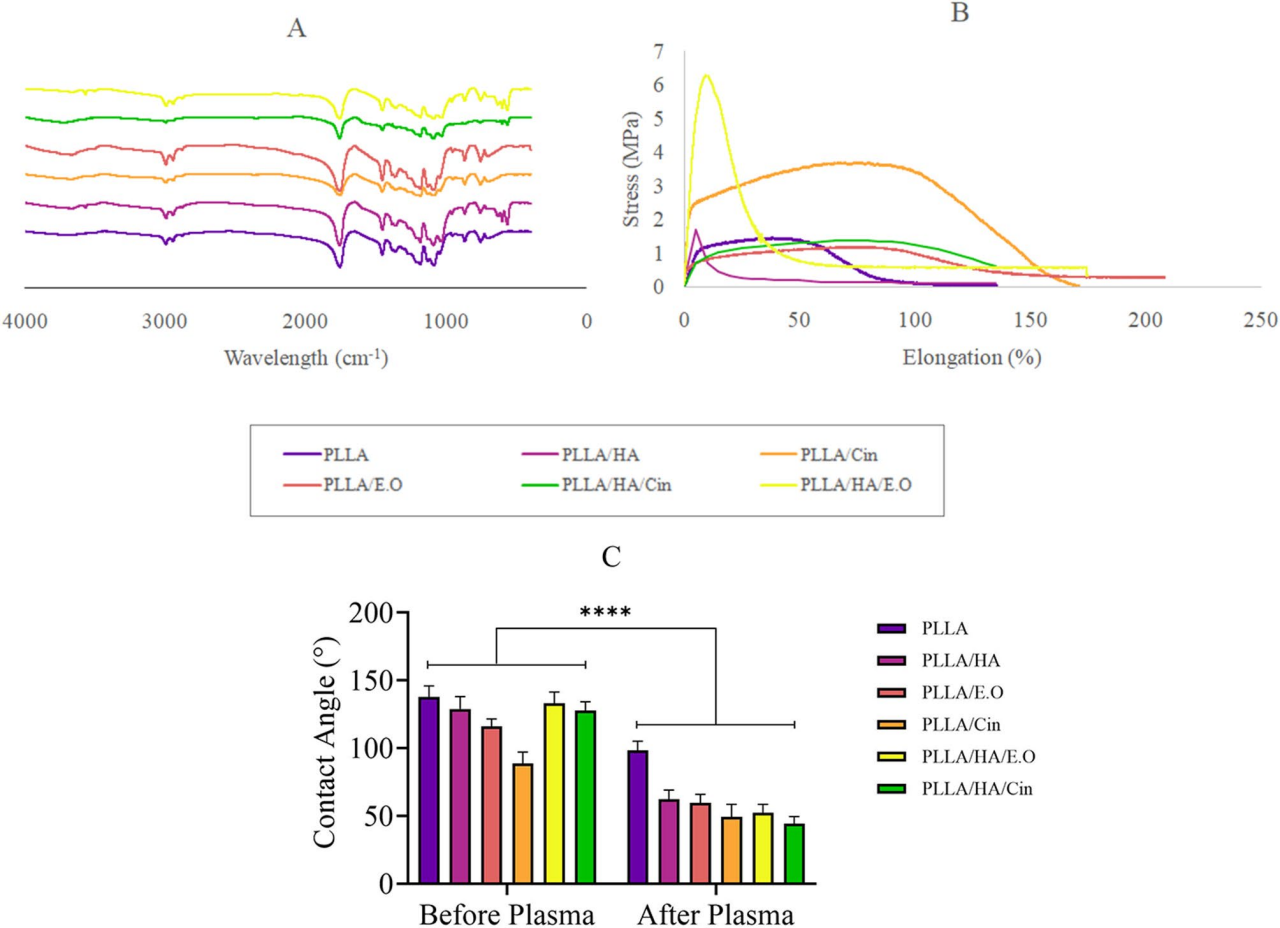
(**A**) FT-IR spectra of different fibrous scaffolds indicate absorption peaks of acidic (1089 cm^− 1^) and esteric (1762 cm^− 1^) groups of PLLA fibrous and absorption peaks of phosphate (570 cm^− 1^, 600 cm^− 1^ and 650 cm^− 1^) groups of HA nanoparticles. (**B**) Tensile strength tested at a strain rate of 10 mm/min indicates that the maximum stress tolerance of PLLA/HA/E.O (6.28 MPa) and PLLA/Cin (3.70 MPa) scaffolds is higher than other fibers. (**C**) Contact angle before and after plasma treatment of PLLA (137.77 ± 8.11°, 98.43 ± 6.61°), PLLA/HA (129.00 ± 9.10°, 62.47 ± 6.64°), PLLA/E.O (116.92 ± 5.59°, 59.69 ± 6.31°), PLLA/Cin (88.83 ± 8.19°, 49.35 ± 9.21°), PLLA/HA/E.O (133.10 ± 8.36°, 52.20 ± 6.41°) and PLLA/HA/Cin (127.76 ± 6.52°, 44.33 ± 5.19°) fibrous scaffolds, respectively. (Mean ± SD, *n* = 3)

FT-IR spectrum analysis in Fig. 2A showed an absorption peak at around 1762 cm^− 1^ and 1089 cm^− 1^ attributed to the stretching vibration of C = O and C-O of ester and acidic groups, respectively in PLLA fibers. Additionally, absorption peaks at around 570 cm^− 1^, 600 cm^− 1^, and 650 cm^− 1^ were attributed to the P-O stretching, *P* = S stretching, and PO_4_ bending of the HA phosphate group respectively in PLLA loaded with HA. The FT-IR spectra of all fibers displayed similar patterns, likely due to the relatively small amount of cinnamaldehyde and essential oil loaded in this study.

The mechanical strength of the fibers was evaluated using a tensile test to assess their resistance to stress imposed by in vitro cell culture conditions [32]. The mechanical strength of each fiber scaffold is shown in Fig. 2B. The incorporation of HA particles or cinnamon-based compounds appeared to enhance the maximum tolerable stress of the composite fiber compared to the pure PLLA fiber.

Although PLLA/HA/Cin (1.40 MPa) and PLLA/E.O (1.18 MPa) fibers showed lower tensile strength compared to PLLA (1.45 MPa) and PLLA/HA (1.67 MPa) fibers, PLLA/HA/E.O. and PLLA/Cinnamaldehyde fibers demonstrated significantly higher tensile strength, with maximum values of 6.28 MPa and 3.70 MPa, respectively, compared to all other fibers. The incorporation of HA particles and cinnamon into PLLA fibers likely increased tensile strength while decreasing the average elongation of PLLA fibers. The matrix of PLLA fibers was stiffer and more elastic due to the presence of HA nanoparticles as an inorganic nanomaterial [11]. Additionally, the tensile strength of cinnamaldehyde- and essential oil-loaded fibers was higher compared to non-loaded fibers. This improvement may result from the polar groups of cinnamaldehyde and cinnamon essential oil forming hydrogen bonds with PLLA chains, thereby enhancing the mechanical properties of the fibers [33].

As shown in Fig. 2C, the surface contact angle of the scaffolds after plasma treatment significantly decreased compared to before treatment. The post-treatment contact angles were as follows: PLLA 98.43 ± 6.61°, PLLA/HA 62.47 ± 6.64°, PLLA/E.O 59.69 ± 6.31°, PLLA/Cin 49.35 ± 9.21°, PLLA/HA/E.O 52.20 ± 6.41° and PLLA/HA/Cin 44.33 ± 5.19°. In contrast, the pre-treatment contact angles were: PLLA 137.77 ± 8.11°, PLLA/HA 129.00 ± 9.10°, PLLA/E.O 116.92 ± 5.59°, PLLA/Cin 88.83 ± 8.19°, PLLA/HA/E.O 133.10 ± 8.36° and PLLA/HA/Cin 127.76 ± 6.52°. These observations indicate an increase in the hydrophilicity of the scaffolding surface. The water contact angle of the scaffold surface depends on the surface chemical composition and topographic configurations of the scaffolds. Enhanced hydrophilicity due to plasma treatment is crucial for tissue engineering applications, as it promotes improved cell attachment and proliferation [34].

### Release profiles of cin and E.O from fibers

**Fig. 3:**
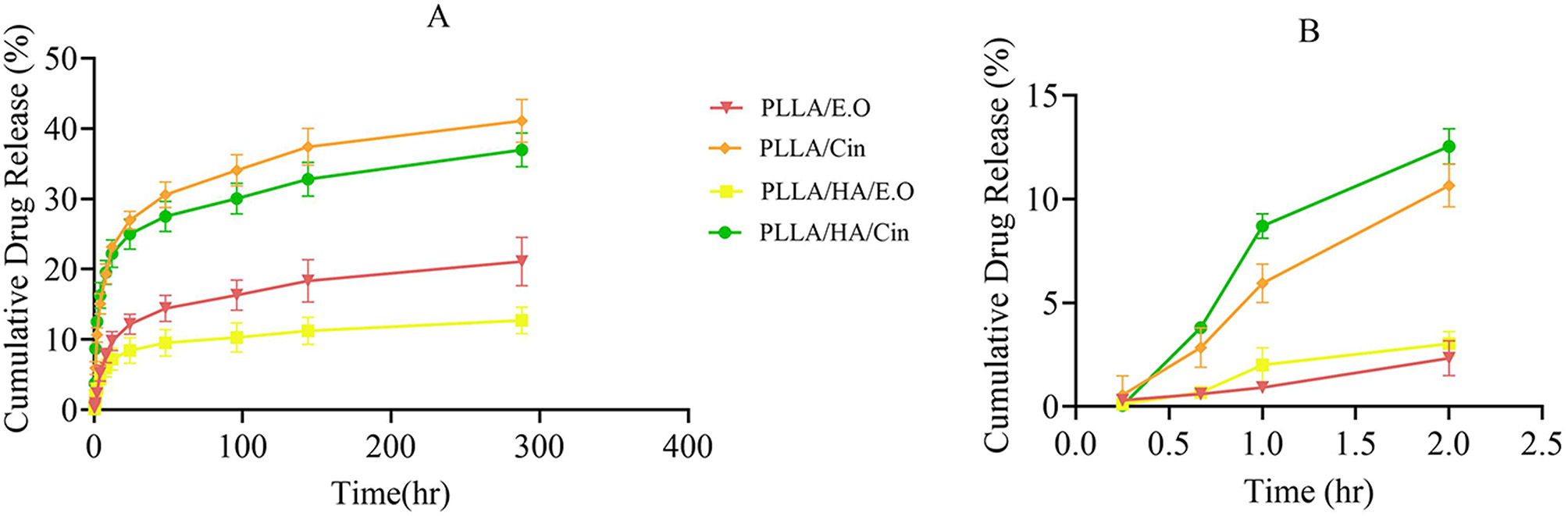
(**A**) Cumulative percentage of Cin and E.O controlled release profile of PLLA/E.O -(21.112 ± 3.433%), PLLA/Cin (41.088 ± 3.049%), PLLA/HA/E.O (12.749 ± 1.914%) and PLLA/HA/Cin (36.981 ± 2.389%) during 12 days at 37° C and pH 7. (Mean ± SD, *n* = 3) (**B**) Cumulative percentage burst controlled release profile of PLLA/E.O (2.325 ± 0.846%), PLLA/Cin (10.657 ± 1.026%), PLLA/HA/E.O (3.035 ± 0.595%) and PLLA/HA/Cin (12.544 ± 0.856%) fibers at first 2 h, 37° C and pH 7. (Mean ± SD, *n* = 3)

To determine the actual Cin and E.O content in the release solution, the release profile of fibers was evaluated over 12 days, as illustrated in Fig. 3. The figure demonstrates the in vitro release profile of Cin and E.O in a two-step biphasic process. Cin and E.O., being highly hydrophobic, tend to resist release after an initial phase [35, 36]. However, Cin and E.O were loaded inside the PLLA matrix, an initial burst release occurred during the first two hours for PLLA/E.O, PLLA/Cin, PLLA/HA/E.O, and PLLA/HA/Cin fibers. The cumulative release during this period was 2.325 ± 0.846%, 10.657 ± 1.026%, 3.035 ± 0.595%, and 12.544 ± 0.856%, respectively (Fig. 3B). This burst release may be attributed to unexpected surface loading of a small amount of Cin and E.O. during the electrospinning process. Following the initial burst, the primary release occurred at a slower, sustained rate over the subsequent 12 days. The cumulative drug release after this period was 21.112 ± 3.433%, 41.088 ± 3.049%, 12.749 ± 1.914%, and 36.981 ± 2.389% for PLLA/E.O, PLLA/Cin, PLLA/HA/E.O and PLLA/HA/Cin, respectively (Fig. 3A).

HA-incorporated fibrous scaffolds exhibited a gentler release profile compared to other scaffolds without HA. Previous studies have shown that the drug release profile in HA-incorporated scaffolds is significantly slower if the drug is loaded within HA nanoparticles. Drug encapsulation into HA particles causes a two-step release that includes drug separation from the HA surface to the matrix and then drug release from the polymer matrix to the external phase solution. As a result, drug release is significantly reduced [37].

In this study, Cin, E.O, and HA particles were loaded into the PLLA matrix. However, the slower release profile of the HA-incorporated scaffold may be attributed to the unexpected loading of a small amount of Cin and E.O. onto HA particles during electrospinning. Additionally, the release profile of Cin-loaded scaffolds showed a significant increase compared to E.O-loaded scaffolds, likely due to established weaker chemical bonds formed between Cin and the PLLA matrix. According to previous studies, essential oils contain different types of chemical compounds with functional groups that may form weak van der Waals interactions with polymer scaffolds [38, 39]. PLLA fibers have a slow-release pattern, making them suitable for prolonged drug release over an extended period [40, 41].

In many clinical cases, an initial medication dose can lead to a rapid achievement of the treatment level. Additionally, continuous drug administration is necessary to maintain these therapeutic levels [42]. Loading drugs into porous structures can increase drug release. In these structures, the drug is released at a higher initial dose and continues at a higher cumulative rate. As a result, the area under the curve increases, indicating the porosity of the fibers [40]. Drug release from electrospun fibers typically occurs through a combination of mechanisms: release of the drug from the fiber surface, diffusion through the pores, and loss of drug binding to the scaffold [43].

In the present investigation, drug release was monitored over a 12-day period to effectively capture both the early and intermediate phases of the delivery profile. Notably, the release profile demonstrated an approach toward a plateau within this timeframe, indicating a substantial completion of the release process. It is common practice in drug release studies involving nanofibers to conclude the analysis once a plateau in release is observed or upon reaching a predetermined endpoint, which may range from 24 h to several weeks depending on the intended application. Therefore, the 12-day monitoring period employed in our study aligns with established protocols and adequately reflects the release kinetics of the system under investigation [44–46].

#### Drug loading and encapsulation efficiency

We have performed an approximate estimation based on the drug release profile and known formulation parameters for drug loading and encapsulation efficiency values (Table 3).

A. Encapsulation Efficiency (EE%):

EE% = (Amount of drug loaded into the scaffold / Total amount of drug initially used) × 100.

B. Drug Loading (DL%):

DL% = (Amount of drug loaded into the scaffold / Total weight of the scaffold) × 100.

##### Assumptions

We assumed that the total drug released over the 12-day period approximately reflects the actual amount of drug encapsulated into the scaffold.

- Initial drug concentrations: 900 µg/mL for cinnamaldehyde, 600 µg/mL for cinnamon essential oil.
- Estimated scaffold weights were ~ 0.00892 g for PLLA/Cin and PLLA/HA/Cin, and ~ 0.00596 g for PLLA/E.O and PLLA/HA/E.O.

**Table 3:** Estimated results

Scaffold Group	EE (%)	DL (%)	Drug Type
PLLA/Cin	41.1%	4.11%	Cinnamaldehyde
PLLA/HA/Cin	37.0%	3.73%	Cinnamaldehyde
PLLA/E.O	21.1%	2.11%	Essential Oil
PLLA/HA/E.O	12.7%	1.27%	Essential Oil

Note: These are approximate values based on cumulative drug release percentages. For more accurate EE and DL measurements, actual drug content in scaffolds should be quantified (e.g., via HPLC or UV analysis after scaffold dissolution)

### Cell adhesion and proliferation

**Fig. 4:**
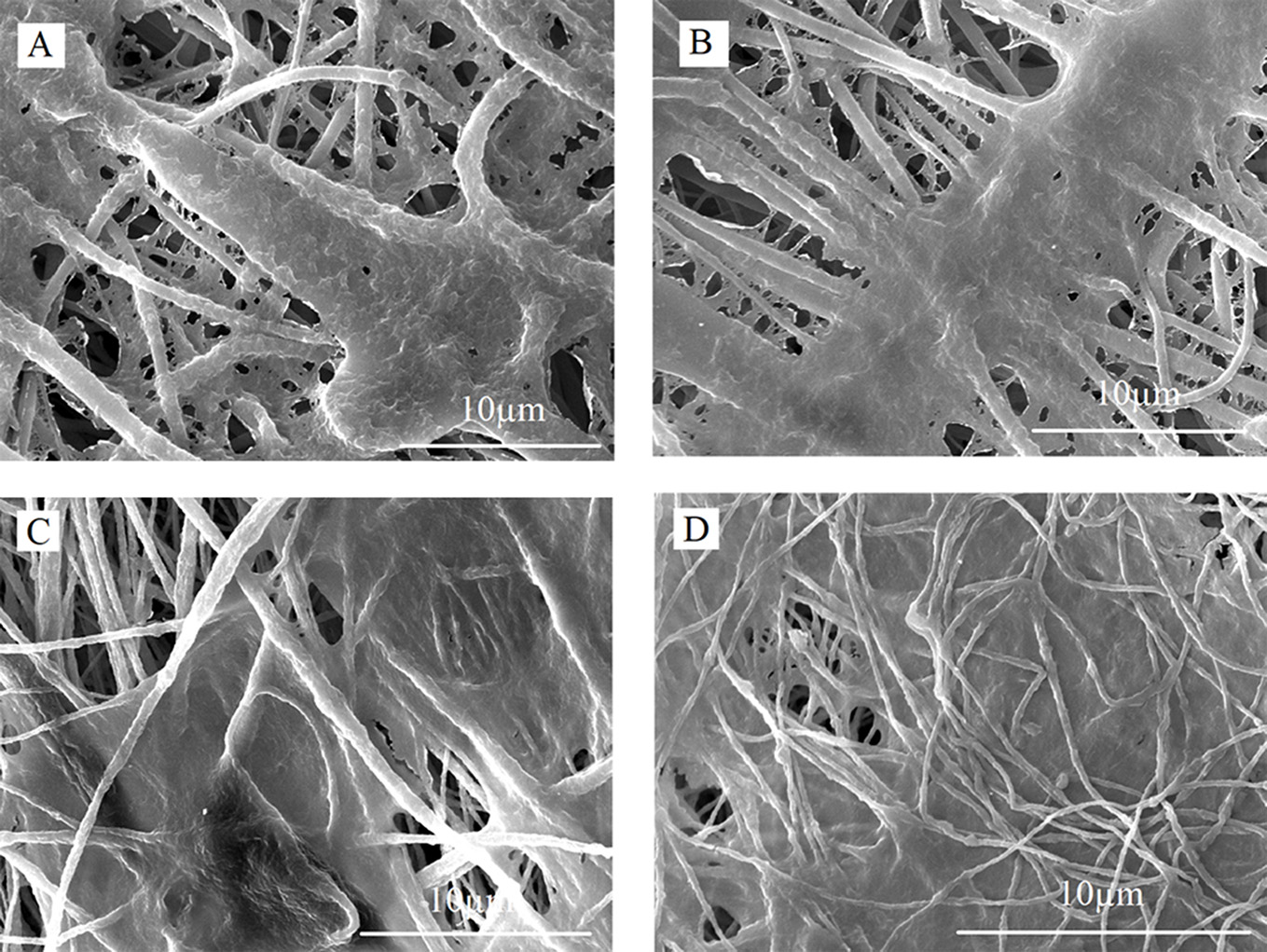
Representative SEM images of Wharton’s jelly MSCs that attached and proliferated to (**a**) PLLA, (**b**) PLLA/HA, (**c**) PLLA/ Cinnamaldehyde, and (**d**) PLLA/HA/ Cinnamaldehyde fibrous scaffolds on day 7

The morphology of Wharton’s jelly cells on PLLA (Fig. 4a), PLLA/HA (Fig. 4.b), PLLA/Cin (Fig. 4c), and PLLA/HA/Cin (Fig. 4d) scaffolds was investigated during cell proliferation on day 7. SEM images showed suitable cell adhesion to the scaffolds (Fig. 4). For bone tissue engineering, a suitable scaffold must support both cell adhesion and proliferation to be able to tissue repair in an in-vitro environment. These properties are critical determinants of the success or failure of scaffolds in promoting osteogenic differentiation [47]. These results demonstrate that the scaffold maintains its structural integrity and supports cellular growth over the tested period. Also, in previous studies, PLLA fibers loaded with hydroxyapatite demonstrated mechanical and thermal stability, indicating their potential suitability as scaffolds for stem cell culture [48–50].

### Cell viability and proliferation analysis

#### Cell viability and IC_50_ of cin and E.O

**Fig. 5:**
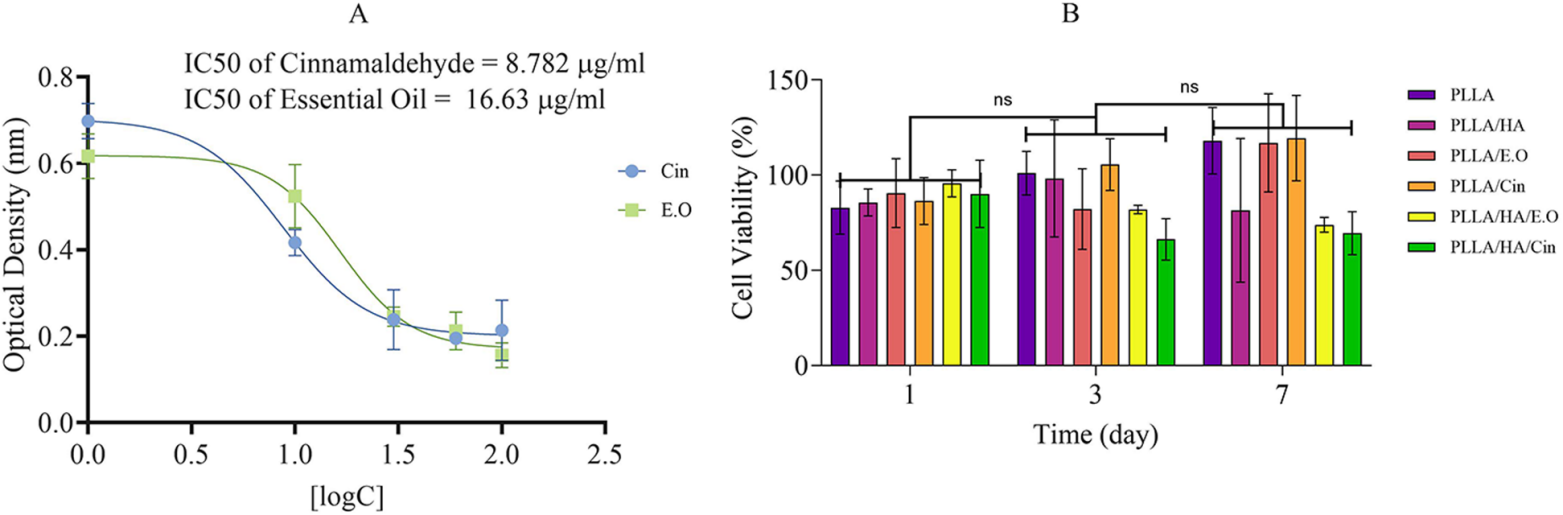
(**A**) Evaluation of *in vitro* cell cytotoxicity by MTT assay for 24 h of cell growth and calculation of Cin and E.O IC_50_ normalized with TCP. (**B**) Evaluation of cell cytotoxicity of fibrous scaffolds by MTT assay that indicates no significant difference between all groups, results normalized with TCP. The tissue culture plate (TCP) group is set as the blank group. Mean ± SD, *n* = 3 (**P < 0.01, ***P < 0.001, ****P < 0.0001)

MTT assay results indicated that less than 50% of Wharton’s Jelly cells survived at concentrations exceeding 37.2 µg/ml and 26.12 µg/ml for Cin and E.O, respectively (Fig. 5A). Therefore, high doses of the Cin and E.O are toxic to Wharton’s Jelly cells. According to other researchers’ studies, Cin can induce cell death by causing apoptosis and mesenchymal-epithelial regression [51]. Additionally, as Cin is an aldehyde, its high toxicity, along with that of E.O., is not unexpected.

#### Cell viability of loaded and non-loaded fibrous scaffolds

MTT results for the scaffolds indicated low toxicity, with no significant differences in cell viability between loaded and non-loaded scaffolds, as shown in Fig. 5B. MTT results for scaffolds were shown that loaded scaffolds did not much toxic in comparison to non-loaded scaffolds. The toxicity of scaffolds did not differ from each other on the first, third, and 7th day of the MTT test. This result indicates that loading Cin and E.O, as toxic natural compounds, into fibers, can lead to their toxicity reduction.

### Differentiation effect of cin and E.O

**Fig. 6:**
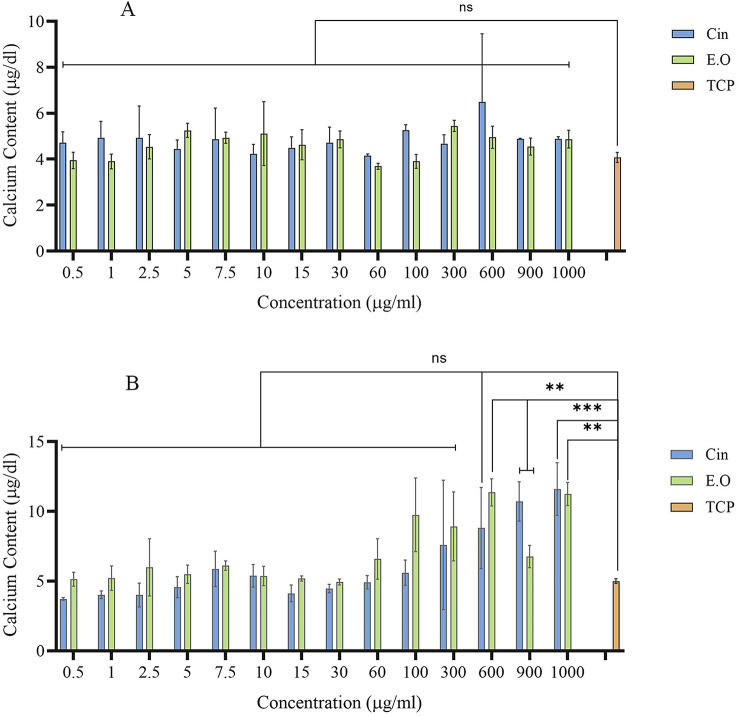
Effect of different concentrations of Cin and E.O on calcium content of MSCs compared with TCP (**A**) after 7 days of cell growth and (**B**) after 14 days of cell growth. The tissue culture plate (TCP) group is set as the blank group. Mean ± SD, *n* = 3 (**P < 0.01, ***P < 0.001, ****P < 0.0001)

The calcium content assay results indicated no significant differences between the groups on day 7 (Fig. 6A). However, on day 14, cell calcium content significantly increased at concentrations of 900 µg/ml and 1000 µg/ml for Cin and 600 µg/ml and 1000 µg/ml for E.O compared to other concentrations and the control group (Fig. 6B).

Osteogenic differentiation of MSCs is a complex process, which leads to the osteoblast production. MSCs differentiation is commonly evaluated by the expression of osteoblast biomarkers. Calcium deposition analysis is a confirmatory test to investigate osteogenic differentiation. Although the MTT assay showed high cell viability at lower concentrations of Cin and E.O, calcium content was significantly enhanced at higher concentrations of Cin (900 µg/ml and 1000 µg/ml) and E.O (600 µg/ml and 1000 µg/ml) compared to the control group. This increase in calcium deposition may indicate the resistance of MSCs to initial stress conditions caused by higher concentrations of Cin. Based on other studies, MSCs can be resistant to stress conditions and then continue to grow and differentiate [52]. Additionally, proliferation and differentiation of cells occur in different phases of the cell cycle; with toxicity primarily affecting the proliferation phase [53].

**Fig. 7:**
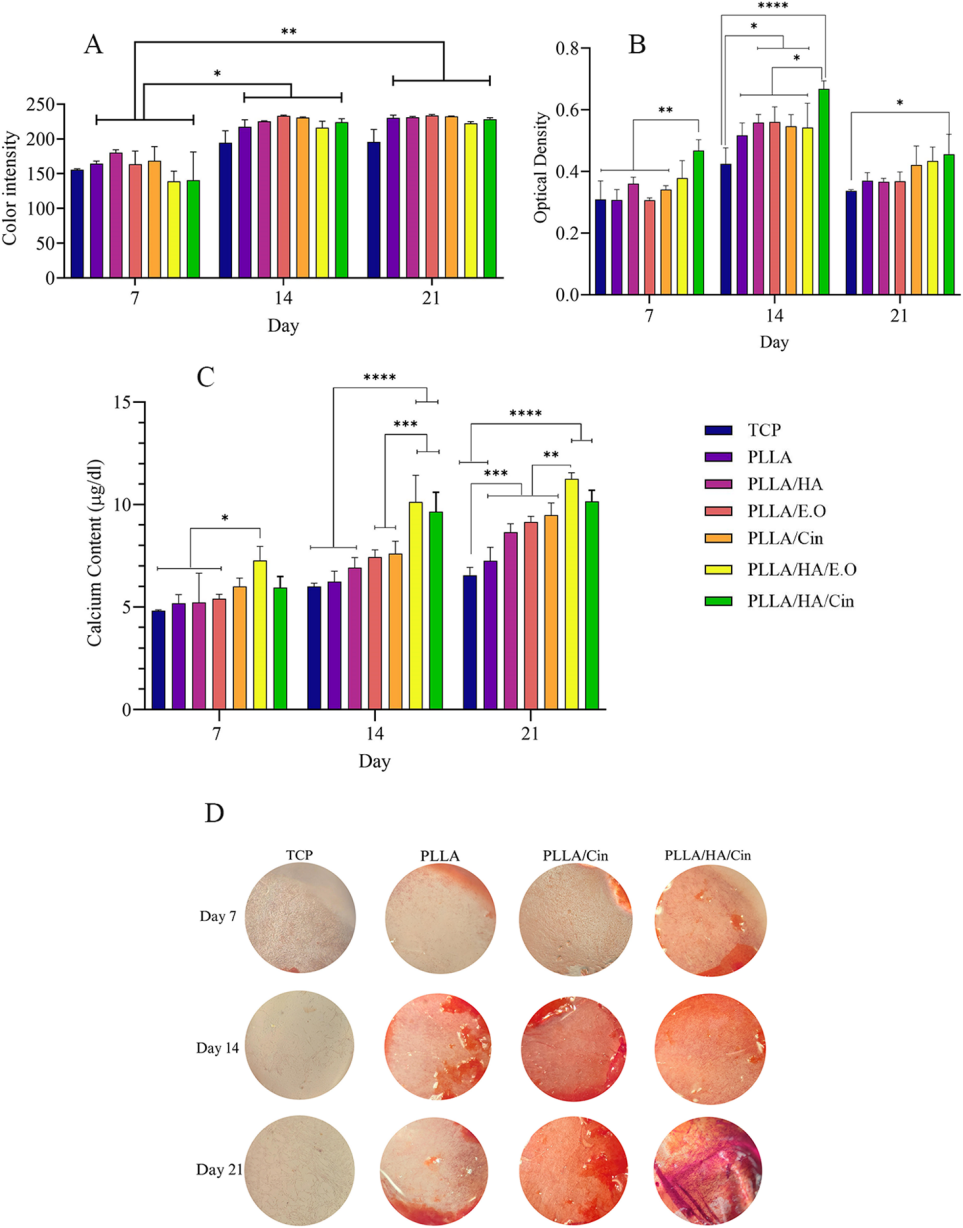
Effect of PLLA, PLLA/HA, PLLA/E.O, PLLA/Cin, PLLA/HA/E.O and PLLA/HA/Cin fibrous scaffolds on (**A**) color intensity of Alizarin red staining, (**B**) Calcium level deposition and (**C**) ALP activity of MSCs after 7, 14 and 21 days of cell growth compared with TCP. (**D**) Selected alizarin red staining images in osteoconductive medium on day 7, day 14 and day 21. The tissue culture plate (TCP) group is set as the blank group. Mean ± SD, *n* = 3 (**P < 0.01, ***P < 0.001, ****P < 0.0001)

**Fig. 8:**
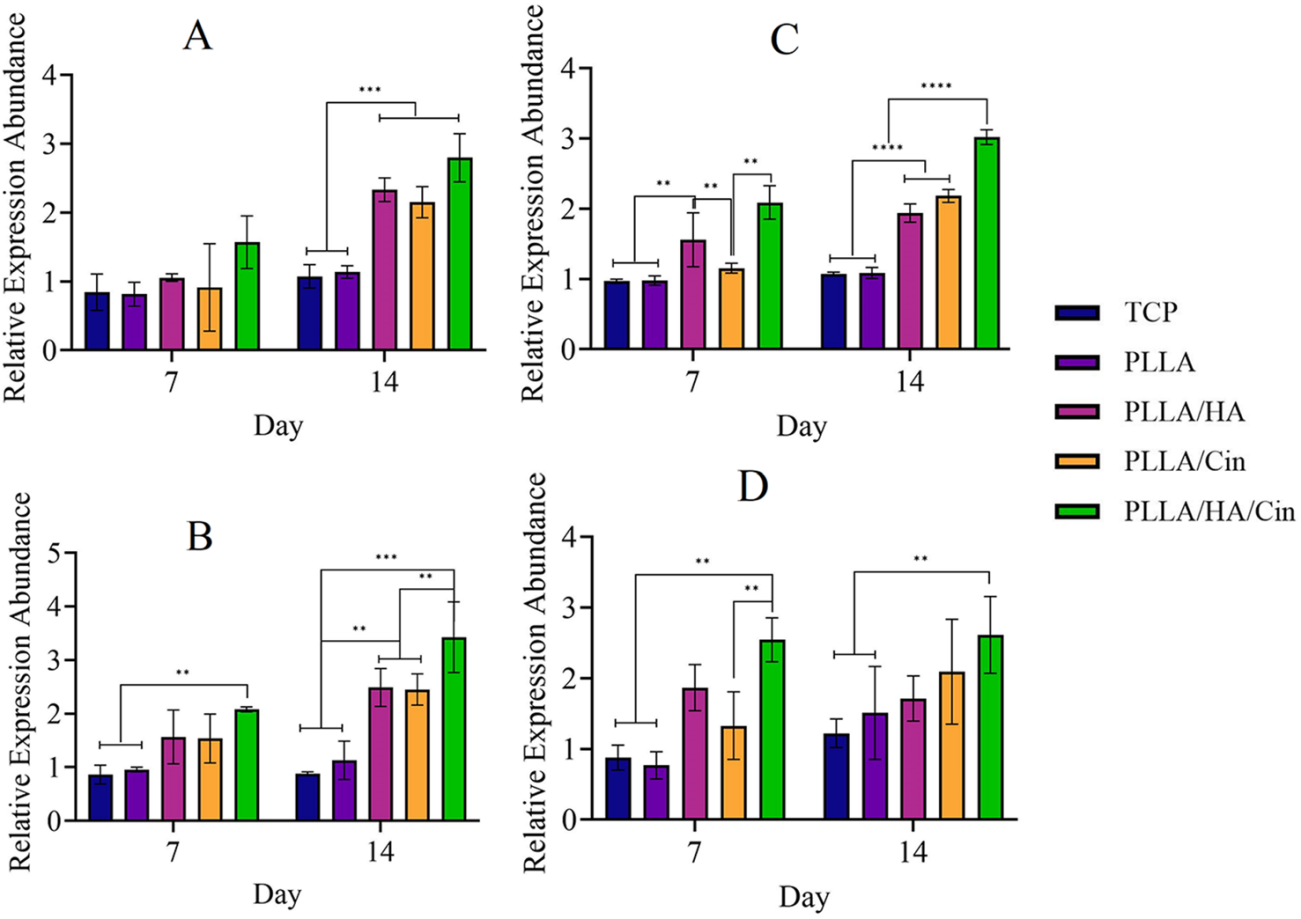
Effect of PLLA, PLLA/HA, PLLA/Cin and PLLA/HA/Cin fibrous scaffolds on relative gene expression (**A**) Runx2, (**B**) Osteocalcin, (**C**) ALP and (**D**) Osteonectin on MSCs after 7 and 14 days of cell growth by PCR real-time test. The tissue culture plate (TCP) group is set as the blank group. Mean ± SD, *n* = 3 (**P < 0.01, ***P < 0.001, ****P < 0.0001)

### Differentiation effect of scaffolds

Osteogenic differentiation of MSCs due to scaffolds’ ability was examined by determining the quantity of bone-related biomarkers such as ALP activity, calcium deposition, and osteogenic-related genes.

Based on the cumulative release profiles, approximately 40% of the total loaded drug (e.g., ~ 360 µg for cinnamaldehyde in PLLA/HA/Cin scaffolds) was released over 12 days. Given the scaffold mass (~ 8.92 mg) and release medium volume (1 mL), the average effective concentration of cinnamaldehyde delivered to the culture environment was estimated to be ~ 36 µg/mL.

This concentration falls within the range previously identified in our preliminary cytocompatibility and osteogenic activity assays (Fig. 6), where cinnamaldehyde at 25–50 µg/mL significantly enhanced calcium content and ALP activity without compromising cell viability.

#### Alizarin red staining study

Figure 7A shows the color intensity of the Alizarin red staining and Fig. 7D shows the selected alizarin red staining images that result was significantly increased on days 14 and 21 in comparison to the color intensity on day 7 for all scaffolds. In addition, the color intensity of the control group was not significant in comparison to the treatment groups.

#### Calcium content study

Calcium deposition level was determined over 21 days to examine osteogenic differentiation. As shown in Fig. 7B, the deposited calcium level was not significantly different between the groups on day 7. However, a similar increasing trend was observed from day 7 to day 14, continuing through day 21. On day 14, calcium level deposition of PLLA/HA/Cin and PLLA/HA/E.O was significantly higher compared to other scaffolds and the control group. However, PLLA/HA/E.O scaffold showed a comparable calcium level deposition with all other groups, but PLLA/HA/Cin showed a calcium level just comparable with the control group and non-loaded scaffolds.

By seeding MSCs on scaffolds, calcium ions are deposited on the surface of the fibers in the presence of dexamethasone, β-glycerophosphate, and ascorbic acid as biomineralization of the late phase of differentiation [10, 54]. Overall, calcium deposition on loaded composite scaffolds was significantly higher than that on non-loaded scaffolds and the control group on days 14 and 21.

#### Alkaline phosphatase study

One of the key indicators of MSCs differentiation is distinguishing the level of ALP activity. Because this biomarker is expressed in the early stages of osteogenesis, therefore, it is commonly used to assess the state of differentiation in the initial days [55]. ALP activity was determined over 21 days to evaluate osteogenic differentiation. As shown in Fig. 7C and consistent with previous studies [56–58] ALP activity had an upward slope from the 7th to the 14th day and then dropped to the 21st for Cin and E.O composited scaffolds compared to other scaffolds and control groups. To justify this phenomenon, it can be mentioned that if alkaline phosphatase continues to increase in the cell membrane, the amount of calcium inside the cells will decrease so much that it will cause cell death [57, 58]. On day 14, the ALP activity of PLLA/HA/Cin and PLLA/HA/E.O significantly increased compared to other scaffolds and the control group. Notably, the PLLA/HA/Cin scaffold showed substantially promoted ALP activity compared to the control group and other scaffolds on day 7, and it demonstrated comparable ALP activity with the control group on day 21. The amount of ALP activity on the 14th day increased in all groups compared to the control group, which may indicate the successful osteogenic differentiation on the surface of the scaffolds.

#### Real-time PCR for gene expression

Differentiation of MSCs into osteoblast cells using scaffolds in the presence of HA and Cin was studied. For quantitative evaluation of osteogenic differentiation, related gene expression including Runx2 (Fig. 8A), osteocalcin (Fig. 8B), ALP (Fig. 8C) and osteonectin (Fig. 8D) was measured and affected by scaffolds for 14 days. All genes showed higher expression on day 14 in comparison to day 7. Although the PLLA/HA/Cin scaffold showed the most effectiveness compared to the PLLA scaffold and control group for osteonectin and Runx2 markers on day 14, it demonstrated a significant differentiation effect than all other groups for osteocalcin and ALP markers on day 14. Additionally, Runx2 marker expression was not significantly different on day 7; other marker expression was different for PLLA/HA/Cin scaffolds into other scaffolds and control group on day 7.

Runx2 is a key transcription factor and an early marker of osteoblast differentiation that regulates the expression of other osteogenic genes. ALP (alkaline phosphatase) is also considered an early marker and plays a crucial role in the initial phase of mineralization by hydrolyzing phosphate esters. Osteocalcin, a late marker of osteogenesis, is a non-collagenous protein secreted by mature osteoblasts and is involved in bone matrix formation and mineral deposition. Osteonectin (also known as SPARC) is a glycoprotein that participates in matrix organization and mineral binding, and it is expressed throughout osteogenic differentiation with increased levels during the intermediate and late stages. These markers together provide a comprehensive view of the progression and maturation of osteogenic differentiation.

### Study limitations and future directions

Despite the promising outcomes demonstrated in vitro, this study has several limitations that should be addressed to support its clinical translation:

#### Absence of in vivo validation

All osteogenic assessments were performed under in vitro conditions using human adipose-derived stem cells (hADSCs). While these results are indicative of potential, they do not fully replicate the complex physiological environment of bone tissue. The lack of in vivo studies means we cannot yet confirm the scaffold’s performance in terms of bone regeneration, host integration, immune response, and vascularization.

Future direction: We plan to conduct animal studies using a critical-size rat calvarial defect model. This will allow us to evaluate not only bone regeneration and mineralization, but also the scaffold’s degradation, inflammatory profile, and integration within host tissue.

#### No long-term degradation analysis

Although mechanical properties and morphology were assessed after 21 days of incubation, longer-term degradation behavior was not studied. PLLA and HA-based scaffolds can exhibit slow degradation kinetics, and prolonged degradation may influence local pH, cell behavior, and structural integrity.

Future direction: We intend to perform long-term in vitro degradation studies (e.g., up to 8–12 weeks), evaluating changes in mass, molecular weight, pH, and mechanical strength. This will help predict in vivo behavior and guide scaffold design for optimal resorption rates aligned with bone healing dynamics.

#### Limited mechanistic insight into osteogenic modulation

While expression of key osteogenic markers was measured, signaling pathways underlying the scaffold-mediated differentiation (e.g., Wnt/β-catenin, MAPK, or BMP signaling) were not explored.

Future direction: Additional molecular studies, including pathway inhibition assays and protein-level validation (e.g., via Western blotting or ELISA), will be incorporated to better understand the mechanistic basis of the observed bioactivity.

#### Scaffold architecture uniformity and pore interconnectivity not evaluated

SEM provided valuable morphological data, but internal porosity and 3D architecture were not quantitatively assessed. These parameters are critical for cell infiltration and nutrient diffusion in vivo.

Future direction: Micro-CT analysis and 3D image reconstruction will be used in future scaffold batches to assess pore architecture, interconnectivity, and spatial distribution of HA.

## Conclusion

In this study, we fabricated dual-function fibrous scaffolds as bone replacement materials. Morphological analysis of the scaffolds revealed appropriate porosity, uniform diameter distribution, and continuous fiber structure. The mechanical property evaluation demonstrated that the incorporation of HA and the loading of Cin and E.O. improved the tensile strength of the scaffolds. Cellular studies showed that while Cin and E.O. exhibited toxicity at high doses, their incorporation into the fibrous scaffolds significantly enhanced the differentiation of mesenchymal stem cells (MSCs) among the tested scaffolds, the electrospun PLLA/HA/Cin scaffold demonstrated superior performance compared to other scaffolds and TCP group. This dual-function system not only supported MSC proliferation but also had a significant effect on stimulating osteoblast differentiation while simultaneously inhibiting osteoclast activity.

## Data Availability

The data that support the findings of this study are available on request from the corresponding author.
